# Genome-Wide Analysis and Screening of Uridine Diphosphate-Glycosyltransferase Family Genes Involved in Lignin/Flavonoid Glycosylation and Stress Response in *Boehmeria nivea* (L.) Gaudich

**DOI:** 10.3390/plants14162517

**Published:** 2025-08-13

**Authors:** Yinghong Tang, Huijuan Tang, Cancai Zhao, Fang Liu, Mingbao Luan, Jianrong Chen

**Affiliations:** 1 Institute of Bast Fiber Crops, Chinese Academy of Agricultural Sciences, Changsha 410205, China; yinghongtang2019@163.com (Y.T.); tanghuijuan@caas.cn (H.T.); 2 College of Life and Environmental Science, Hunan University of Arts and Science, Changde 415000, China; 19330295292@163.com; 3 Changde Synthetic Biology Manufacturing Industry Innovation Center, Changde 415000, China; 4 College of Biological and Chemical Engineering, Changsha University, Changsha 410022, China; lf800825@163.com; 5 National Nanfan Research Institute (Sanya), Chinese Academy of Agricultural Sciences, Sanya 572000, China; 6 National Center of Technology Innovation for Comprehensive Utilization of Saline-Alkali Land, Dongying 257347, China

**Keywords:** *Boehmeria nivea*, uridine diphosphate-glycosyltransferase, lignin, flavonoid, expression and correlation analysis, cadmium stress

## Abstract

Lignins and flavonoids, which are derived from the phenylpropanoid pathway and share common precursors, play an important role in *Boehmeria nivea* (ramie). Uridine diphosphate-glycosyltransferases (UGTs) are essential for the glycosylation of secondary metabolites and are involved in plant growth and stress responses. Hence, this study aimed to screen candidate *UGTs* related to lignin/flavonoid glycosylation and stress responses. A total of 84 *BnUGT*s were identified, and all *BnUGT*s contain a conserved PGPS domain. Phylogenetic analysis suggested that 10, 5, 1, and 1 putative *BnUGT*s might be associated with lignin glycosylation, flavonoid glycosylation, and adverse stress, respectively. Further analysis showed that *Bnt05T007753.1* expression was upregulated and showed a significant positive correlation with lignin content in the phloem and leaf, reaching up to 710 in the xylem after 75 days of germination. *Bnt14T019888.1* expression (in the leaf and xylem) and *Bnt06T010117.1* expression (in the xylem) were upregulated and showed a significant positive correlation with lignin and flavonoid content. In the phloem, *Bnt14T019888.1* expression was downregulated and showed a significant negative correlation with lignin content. *Bnt04T006105.1* expression was upregulated in the stem and leaf under Cd treatment. Overall, we successfully identified four candidate *BnUGTs* (*Bnt05T007753.1*, *Bnt14T019888.1, Bnt06T010117.1*, and *Bnt04T006105.1*); these findings provide insight into the glycosylation mechanisms of lignins and flavonoids and stress responses in ramie.

## 1. Introduction

**Figure 1:**
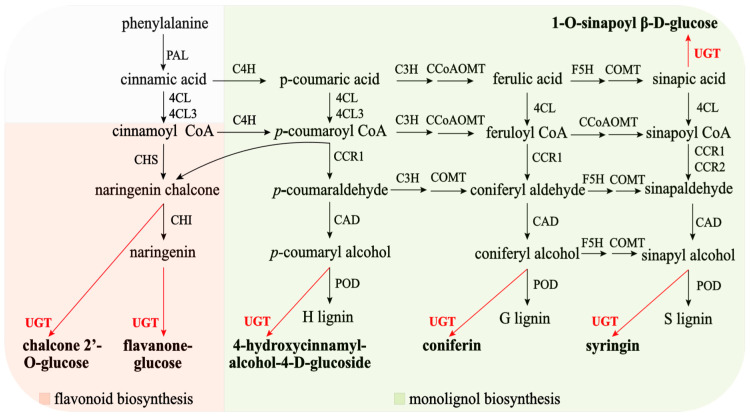
The monolignol biosynthesis pathway and flavonoid biosynthesis pathway with associated glycosylated products in ramie [6]. PAL: Phenylalanine Ammonia-Lyase; 4CL: 4-Coumarate-CoA Ligase; C4H: Cinnamate-4-Hydroxylase; C3H: Coumarate-3-Hydroxylase; CCoAOMT: Caffeoyl-CoA-3-O-Methyltransferase; CCR: Cinnamoyl-CoA Reductase; COMT: Caffeic Acid-O-Methyltransferase; F5H: Ferulate-5-Hydroxylase; CAD: Cinnamyl Alcohol Dehydrogenase; POD: Peroxidase; UGT: Uridine diphosphate glycosyltransferase; CHS: Chalcone synthase; CHI: Chalcone isomerase; H lignin: p-Hydroxy-phenyl lignin; G lignin: Guaiacyl lignin; S lignin: Syringyl lignin.

*Boehmeria nivea* (L.) Gaudich. (ramie) is a bast fiber crop used in textiles, medicine, feed, and environmental protection, making it a valuable alternative plant [1,2]. Lignins play an important role in plant defense and development and are a critical factor affecting the fiber and quality of ramie feed. Flavonoids are also important components of ramie feed and have medicinal value. Both lignins and flavonoids are formed via the phenylpropanoid pathway and share common precursors. Uridine diphosphate-glycosyltransferases (UGTs, EC 2.4.1.x) are a large family of enzymes that catalyze the transfer of sugar to a variety of plant secondary metabolites involved in lignan, flavonoid, salicylate, and phytohormone metabolism. This has potential implications for cell wall biosynthesis and biotic and abiotic stress responses [3]. Plant UGTs play diverse roles at different developmental stages, and most of their substrates are derived from the phenylpropanoid pathway, whose precursors are the aromatic amino acids phenylalanine and tyrosine [4,5]. Therefore, this study focused on *UGT* genes involved in monolignol and flavonoid biosynthesis and their abiotic stress responses in ramie (Figure 1).

Monolignols (or (hydroxyl)cinnamyl alcohols) such as *p*-coumaryl alcohol, coniferyl alcohol, and sinapyl alcohol are the main building blocks of lignins. In plants, monolignol glycosylation is catalyzed by family 1 UGTs. When glycosylated by UGTs in the presence of uridine diphosphate (UDP)-glucose, these phenylpropanoids give rise to cinnamyl alcohol glucosides, coniferin, and syringin. In ramie, UGTs can also catalyze the glycosylation of sinapic acid (Figure 1). Monolignol glucosides accumulate in vascular tissues, including the phloem, cambial tissue, and differentiating xylem, of both conifers and angiosperms and can be incorporated into lignin polymers [7]. Monolignol glycosylation catalyzed by *UGT* is essential for lignification; however, the role of *UGT* genes in ramie lignin synthesis has not been well investigated. Most studies have focused on lignin biodegradation mechanisms, which are vital factors responsible for lignin modification and degradation [8]. Monolignol glycosylation plays a key role in lignin metabolism in *Arabidopsis thaliana* (L.) Heynh, poplar, *Ginkgo biloba* L. [9], and *Pyrus bretschneideri* Rehder. This is carried out via *UGT72E* [10] and *UGT72B* [11] subfamilies in *Arabidopsis thaliana* and *UGT72AZ1*, *UGT72AZ2*, *UGT72B37*, and *UGT72B39* in poplar [7] glycosylate monolignols. *Arabidopsis* and poplar exhibit partial conservation of substrate recognition between *UGT72*, and divergent functions exist between different groups of the *UGT72* family [7]. *PbUGT72AJ2* of *P. bretschneideri* is considered a monolignol-glycosylation-related UGT [12]. In *Arabidopsis*, the guaiacyl (G) unit is 2–3 times more abundant than the syringyl (S) unit [13]. The lignin in poplar mainly consists of G units, with a minor amount of H (hydroxyphenyl) units [14]. The ratio of S, G, and H units in ramie lignin is approximately 6:3:1 [15], which differs from that in the model plant. Therefore, research on *UGT* genes in model plants has greatly promoted the study of monolignol glycosylation in ramie and other fiber crops.

Flavonoids are secondary plant metabolites that are widely distributed in nature. They contain at least 10,000 different derivatives with excellent biological functions. The biotransformation of flavonoid aglycones into *O*-rutinosides or *O*-neohesperidosides in *Citrus* plants usually involves two glycosylation reactions involving a series of uridine diphosphate-sugar-dependent glycosyltransferases [16]. Complementary DNAs (cDNAs) encoding flavonoid diglycosyltransferases from *Citrus* have been identified and functionally characterized. These cDNAs include those encoding flavanone/flavone-7-O-glucoside-1,2-rhamnosyltransferase from pummel [17] and *CsUGT76F1* from *Citrus sinensis* (L.) Osbeck [16]. Similarly, Chen et al. [18] identified a flavonoid (1–2 rhamnosyltransferase) involved in flavanone neohesperidoside biosynthesis. UGTs are a group of enzymes responsible for the glycosylation of flavonoid glycosides in *Epimedium pubescens* Maxim. [19]. In *Camellia sinensis* (L.) Kuntze, two *CsUGT* genes (*CsUGT75L12* and *CsUGT79B28*) participate in the biosynthesis of the bitter flavonoid 7-O-neohesperidoside via sequential glycosylation and rhamnosylation of flavonoids [20]. *OsUGT88C3* is responsible for the biosynthesis of malvidin 3-*O*-galactoside in rice [21]. *RchUGT169* can catalyze the conversion of kaempferol and quercetin to the corresponding flavonoid glycosides in *Rubus chingii* Hu through transient expression in tobacco [22]. In ramie, hydroxycinnamoyl CoA ester is a precursor for *UGT* to form chalcone glucose and flavanone glucose (Figure 1). However, the role of *UGT* genes in plant flavanone synthesis has not been thoroughly investigated. Therefore, studying *UGT* genes in ramie is necessary.

Despite the widespread identification of *UGT* family members in various species, limited information is available on this family in ramies. Therefore, a genome-wide analysis was conducted in this study to identify *UGT* family members in the *B. nivea* genome. This resulted in the identification of 84 *UGT* genes. These *UGT* genes were systematically characterized, including their phylogenetic relationships, gene structures, conserved motifs, cis-acting elements, chromosome distribution, and gene duplication events. Importantly, *UGT* genes involved in monolignol and flavonoid biosynthesis were identified and characterized. Moreover, expression profiles and accumulation of lignin and flavonoids in ramie during growth were evaluated. In addition, the abiotic stress responses of *UGT* genes involved in monolignol and flavonoid biosynthesis in ramie were validated. Therefore, comprehensive genome-wide identification and rigorous characterization of these *UGT* genes in ramie are important for understanding the glycosylation mechanisms and regulation of monolignol and flavonoid accumulation in the plant. Thus, this study provides valuable insights into the further functional characterization of *UGT* genes in ramie.

## 2. Results

### 2.1. Identification and Characterization of BnUGT Genes

Based on 90 *Arabidopsis UGT* amino acid sequences, 84 *UGT* genes were identified in *B. nivea* (*BnUGT*) based on the presence of complete plant secondary product glycosyltransferase (PSPG) motifs. Further characterizations, including protein lengths, MW, pI, GRAVY, index values, and subcellular localization, are presented in Supplementary Table S1. Protein lengths varied from 119 to 768 amino acids, corresponding to their MWs ranging between 13,162.84 and 84,954.12 Da, and the pI changed from 4.95 to 8.90. The instability index changed from 32.01 to 74.07, the aliphatic index changed from 64.87 to 106.15, and GRAVY changed from −0.533 to 0.132. The above data indicate that the physical and chemical properties of the 84 *BnUGT* proteins showed substantial differences. Subcellular localization predictions indicated that the cell membrane was the primary cell localization structure for these UGT proteins, followed by the chloroplasts. Notably, two *BnUGT* proteins (*Bnt08T012480.1* and *Bnt13T018476.1*) were observed over five localization structures.

### 2.2. Phylogenetic and Classification Analysis of BnUGT Genes

**Figure 2:**
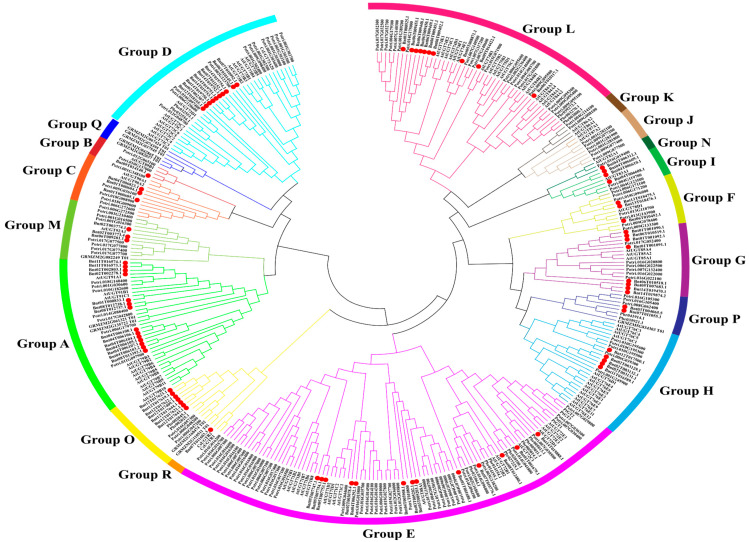
Interspecific phylogenetic tree of UGT protein sequences. Different colors represent different groups. Red circle represents *UGT* genes of ramie.

**Figure 3:**
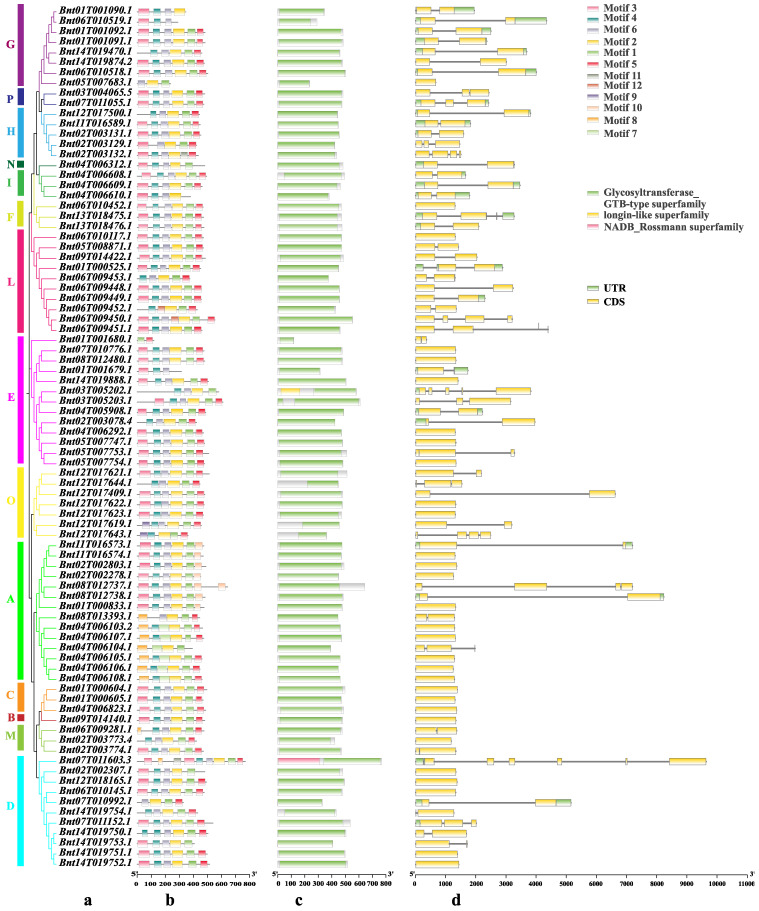
The gene structures, conserved motifs, and conserved domains of *BnUGT* family members based on the evolutionary relationship. (**a**) Phylogenetic tree based on the full-length protein sequences of 84 *BnUGTs*; (**b**) conserved motifs of *BnUGT* proteins; (**c**) conserved domain of BnUGT proteins; (**d**) exon–intron structure of *BnUGT* proteins. *UGTs* from various groups are represented in different colors.

To classify and better understand the evolutionary relationships among *BnUGT* proteins (Supplementary Table S2), a phylogenetic tree of *BnUGT* genes and other known *UGT* proteins from various plants was constructed (Supplementary Table S3). In total, 84 *BnUGT* genes in groups A–I, L–P, and R were found (Figure 2 and Figure 3a). The study focus was to screen the members of phylogenetic groups involved in monolignol and flavonoid biosynthesis.

The *UGT* genes of Group A generally use anthocyanin as the substrate [23], and *ZmUGT* genes from *Z. mays* (*GRMZM2G135722_T01* and *GRMZM2G061321_T01*) in Group A all encode anthocyanidin 3-O-glucosyltransferase [24]. Thus, the 14 *BnUGTs* (such as *Bnt04T006105.1*) in this phylogenetic group are likely associated with the glycosylation of anthocyanins. *AtUGT89B1* in Group B is mainly responsible for glycosylation of dihydroxybenzoic acids and flavonoids [25]. *UGT* genes in Group F can glycosylate anthocyanidins and flavonols [23]. In the phylogenetic tree constructed in this study, *AtUGT78D1* (flavonol 3-O-glycosyltransferases) in Group F catalyzes the formation of anthocyanidin or flavonol glycosides [26]. Therefore, the fourteen *BnUGT* genes in Group A, *Bnt09T014140.1* in Group B, and the three *BnUGT* genes (*Bnt06T010452.1*, *Bnt13T018475.1*, and *Bnt13T018476.1*) in Group F may catalyze flavonoids to form glycosides.

The *UGT* genes responsible for the glycosylation of monolignols and lignin precursors are mainly distributed in Groups D, E, and L [27]. *AtUGT73C5* in Group D catalyzes the formation of cinnamyl alcohol glucoside [28], and *Pbr032554.1* and *Pbr032553.1* are associated with glycosylation of monolignols or lignin precursors [12]. The phylogenetic relationships of *BnUGT* genes in the same clade, such as *Bnt12T018165.1*, which is closest to *AtUGT73C5*, *Pbr032554.1*, and *Pbr032553.1*, suggest that this gene may be associated with monolignol glycosylation. *CsUGT73A20* (*C. sinensis*) in Group D catalyzes the formation of flavonol glycosides [29], and *CtUGT49* (*Carthamus tinctorius* L.), which belongs to the *UGT73* family, catalyzes the conversion of naringenin chalcone [30]. Thus, *BnUGT* genes in the same clade, such as *Bnt07T010992.1*, which is closest to *CsUGT73A20*, may have similar functions.

The highest number of UGTs that catalyze the formation of monolignol/lignin precursor glucose esters or glucosides is in Group E. *AtUGT71C1* catalyzes the glycosylation of caffeic acid [31], and *Potri.016G014500* was maximally down-regulated under drought conditions. Moreover, five *BnUGT* genes (*Bnt02T003078.4*, *Bnt04T006292.1*, *Bnt05T007747.1*, *Bnt05T007753.1*, and *Bnt05T007754.1*), which are closest to *AtUGT71C1* in the phylogenetic tree, may also be associated with monolignol glycosylation and adverse stress. Among them, *AtUGT72E2* simultaneously catalyzes the glycosylation of lignin monomers (coniferyl alcohol and sinapyl alcohol) [32] and precursors (coniferyl aldehyde, sinapyl aldehyde, and sinapic acid) [10], and *AtUGT72D1* catalyzes the glycosylation of sinapic acid [33]. The phylogenetic relationships of *Bnt14T019888.1* and *Bnt01T001679.1* were the closest to *AtUGT72E2* and *AtUGT72D1*. This relationship confirmed that these genes may catalyze similar substrates. *AtUGT72B1* catalyzes the glycosylation of coniferyl aldehyde and alcohol and regulates cell wall development and lignification. Notably, *AtUGT72B1* alters the total amount of lignin in plants [11]. *Potri.014G096100*, *Potri.002G168600* [7], *Pbr014154.1*, and *Pbr014155.1* [12] were considered to be monolignol-glycosylation-related *UGT* genes, and the phylogenetic relationships of *Bnt08T012480.1*, *Bnt07T010776.1*, and *Bnt01T001680.1* were closest to *AtUGT72B1*, *Potri.014G096100*, *Potri.002G168600*, *Pbr014154.1*, and *Pbr014155.1*. Therefore, it was hypothesized that these *BnUGT* genes catalyze glucose conjugation of monolignols.

Members of Group L identify the carboxyl groups of different metabolites, such as phenylpropanoids and auxins [25]. *AtUGT75C1* in Group L was identified as anthocyanin 5-O-glucosyltransferases from various plant species. *UGT75C1* is functionally non-redundant in *A. thaliana* because its mutant (ugt75c1) completely lacks anthocyanin 5-O-glucosides associated with flavonoid metabolism [34]. *AtUGT84A1*, *AtUGT84A2*, *AtUGT84A3*, and *AtUGT84A4* in Group L catalyze the formation of cinnamic acid and hydroxycinnamic acids (*p*-coumaric acid, caffeic acid, ferulic acid, and sinapic acid) from lignin precursor glucose esters [35,36]. This study hypothesized that the ten *BnUGT* genes in the same clade can also participate in flavonoid and monolignol metabolism. The study focused on *Bnt06T010117.1* because it was clustered in the phylogenetic tree of *AtUGT75C1* and *AtUGT84A1-4*.

In addition, Groups O and P are newly discovered taxa in higher plants; seven members were observed in Group O, and two were observed in Group P. *GRMZM2G110511_T01* and *GRMZM2G168474_T02* in Group O are cis-zeatin *O*-glucosyltransferases, and *GRMZM2G082249_T01* (Group M) and *GRMZM5G834303_T01* (Group P) are cytokinin-*O*-glucosyltransferases [3,24]. Members of these three groups may be closely associated with plant hormone glycosylation. Therefore, this study hypothesized that the *BnUGT* genes in groups M, O, and P may be associated with hormone glycosylation. In addition, *Potri.016G105400* from the newly formed phylogenetic Group P was found to be upregulated under drought stress, thereby suggesting that *Bnt03T004065.5* and *Bnt07T011055.1* in Group P may be associated with adverse stress. Moreover, ramie contained one Group R member (*Bnt07T011603.3*) but with the absence of Group Q (Supplementary Table S4).

Based on the phylogenetic tree, seventeen *BnUGTs* were screened, including ten putative lignin-glycosylation-related *BnUGT*s (*Bnt07T010992.1*, *Bnt12T018165.1*, *Bnt02T003078.4*, *Bnt04T006292.1*, *Bnt05T007747.1*, *Bnt05T007753.1*, *Bnt05T007754.1*, *Bnt14T019888.1*, *Bnt08T012480.1*, and *Bnt06T010117.1*); five putative flavonoid-glycosylation-related *BnUGT*s (*Bnt04T006105.1*, *Bnt09T014140.1*, *Bnt06T010452.1*, *Bnt13T018475.1*, and *Bnt13T018476.1*); one putative adverse-stress-related *BnUGT* in newly formed phylogenetic Group P (*Bnt03T004065.5*); and one putative plant-hormone-related *BnUGT* (*Bnt02T003773.4*). Further analysis was conducted on the expression of these putative *BnUGTs* in different tissues during the growth and development of ramie and their response to cadmium stress.

### 2.3. Gene Structure, Conserved Motif, and Conserved Domain Analysis of BnUGT Genes

The motif composition of *BnUGT* proteins is shown in Figure 3b and Supplementary Figure S1, yielding 12 conserved motifs specific to ramie *UGT* proteins. Motif 1 (78/84), motif 2 (81/84), and motif 4 (78/84) were universally present across all *BnUGT* proteins, thus representing the conserved *UGT* domain. Only *Bnt01T001679.1* (314 aa), *Bnt01T001680.1* (119 aa), and *Bnt06T010519.1* (289 aa) did not contain motif 2, which might be responsible for the short length of the protein sequence. Using SMART to annotate each motif, only motifs 1 and 2 were annotated as uridine diphosphate-glucuronosyltransferase domains (PF00201), and motif 1 contained the complete PSPG-box. Notably, the distribution of some motifs displayed subgroup specificity. For example, the motif composition was divided into two parts in Group A, which is consistent with the evolutionary tree relationship. Members of the same group have similar motifs, which is consistent with the evolutionary relationships between members. Cluster analysis based on similar motif compositions revealed potential functional similarities among *BnUGT* proteins. The conserved domains of *BnUGT* proteins are shown in Figure 3c and Supplementary Table S5. All *BnUGT* proteins contain a glycosyltransferase GTB-type superfamily domain. The presence of a conserved PGPS domain within the obtained protein sequences was confirmed.

Additionally, the gene structures of *BnUGT* family members were obtained. The genes exhibited a diverse range of intron–exon arrangements, and most *BnUGT* genes consisted of introns and exons (Figure 3d). The number of exons in *BnUGT* genes varies from one to six. A total of 28 members were intronless genes, and Groups H, I, N, and P did not contain intronless genes. In combination with gene structure and phylogenetic tree analyses, the patterns of exon–intron distribution between *BnUGT* genes of the same phylogenetic group were very similar. This phenomenon further revealed the closer evolutionary relationship between these members and simultaneously showed the reliability of the phylogenetic tree construction.

### 2.4. Chromosomal Distribution and Gene Collinearity Analysis of BnUGT Genes

**Figure 4:**
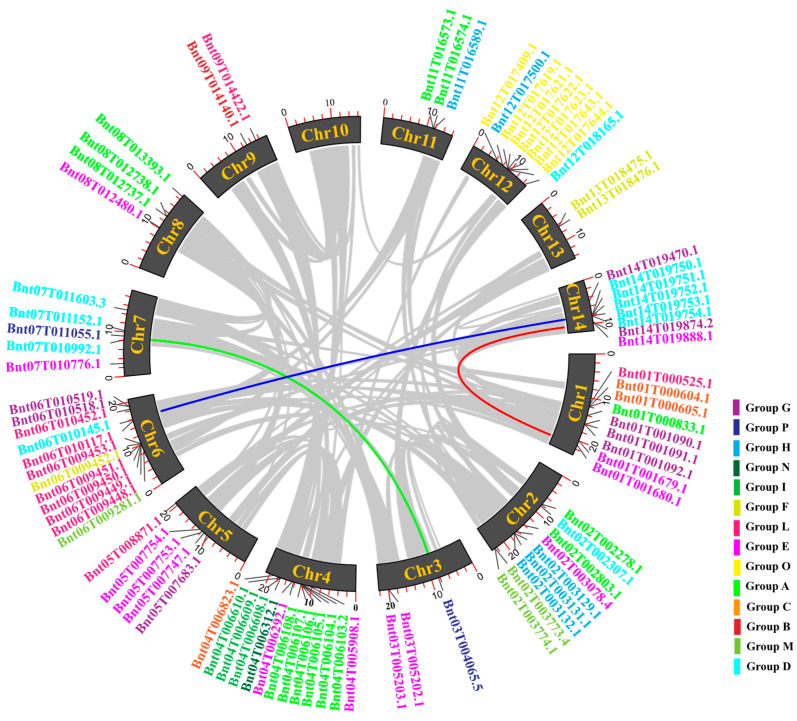
The chromosome distribution information and *gene collinearity* of *UGT* genes within ramie sinensis. Gray lines represent all synteny gene pairs in the ramie genome, and chromatic color lines represent WGD or segmental duplicates of *BnUGT* gene pairs. *UGTs* from various groups are represented in different colors.

In total, 84 *BnUGT* genes were assembled and located on chromosomes 1–14 of ramie (except for chromosome 10) (Figure 4). Among the chromosomes, chr4 and chr6 harbored the highest number of *BnUGT* genes (13/12). Chr1, chr2, and chr12 harbored the same number of *BnUGT* genes, all containing nine genes. Two members were observed on chromosomes 9 and 13. The number of *BnUGTs* on chromosomes 9 and 13 was minimal, and there were three *BnUGTs* on chromosomes 3 and 11. The distribution of different phylogenetic group members on the chromosomes was irregular and mostly existed as gene clusters. The Group O members were all on chromosome 12, while the *BnUGTs* on chromosomes 1, 2, 4, and 6 were from five phylogenetic groups. A total of six members of Group A and Group L formed gene clusters on chromosomes 4 and 6, respectively. The members of Group E were mainly distributed on chromosomes 1, 2, 3, 4, 5, 7, and 8; the members of Group A were mainly distributed on chromosomes 1, 2, 4, 8, and 11; and the members of Group D were mainly distributed on chromosomes 2, 6, 7, 12, and 14. In addition, the results suggest that there were three collinear gene pairs in the study: *Bnt01T001679.1* demonstrated a collinear relationship with *Bnt14T019888.1*; *Bnt03T004065.5* demonstrated a collinear relationship with *Bnt07T011055.1*; and *Bnt14T019754.1* demonstrated a collinear relationship with *Bnt06T010143.2*. Comprehensive analysis of collinearity and gene duplication events demonstrated the expansion and diversification of *BnUGT* gene members in ramie.

### 2.5. Analysis of Cis-Acting Elements of Ramie UGT Genes

A total of thirty cis-acting elements were recognized from the promoter region of the *BnUGT* genes, which were grouped into seven classes, including nine light-responsive elements (44.88%), seven defense and stress-responsiveness elements (12.66%), four phytohormone-responsive elements (25.49%), four MYB binding sites, four protein binding sites, one auxin-responsive element (8.21%), and one flavonoid biosynthetic binding site (Supplementary Table S6).

Among all the elements, the G-box had the highest frequency (11.56%), followed by Box4 (10.84%). Additionally, methyl jasmonate response elements (134 CGTCA-motif and 133 TGACG-motif), abscisic acid response elements (218 ABRE), gibberellin-responsive elements (28 P-box, 17 GRAR-motif, and 17 TCTC-box), and salicylic acid response elements (48 TCA-element, 3 SARE, and 1 MRE) were detected in *BnUGT* genes. Six stress-responsive elements, including ARE (176), LTR (49), TC-rich repeats (40), MBS (29), GC-motif (20), and WUN-motif (4), were retrieved from ramie UGTs and responded to anaerobic induction, low-temperature induction, defense and stress prompts, drought, anoxic induction, and wounding stresses. Several plant-growth-related elements, such as the CAT-box (45), GCN4-motif (17), circadian (13), RY-element (6), MSA-like (3), HD-Zip1 (2), and AACA_motif (1), were obtained from ramie *UGT* genes. These elements are involved in meristem and endosperm expression, circadian control, seed-specific regulation, cell cycle regulation, palisade mesophyll cells, and negative endosperm-specific expression. Moreover, cis-acting elements associated with flavonoid biosynthetic gene regulation and four MYB-binding sites were identified within the *BnUGT* promoter sequences. The composition of these cis-acting elements showed that most *BnUGT* genes were involved in light, stress, hormones, and plant development.

### 2.6. The Analysis of the Lignin Content, Flavonoid Content, and Putative BnUGT Gene Expression

**Figure 5:**
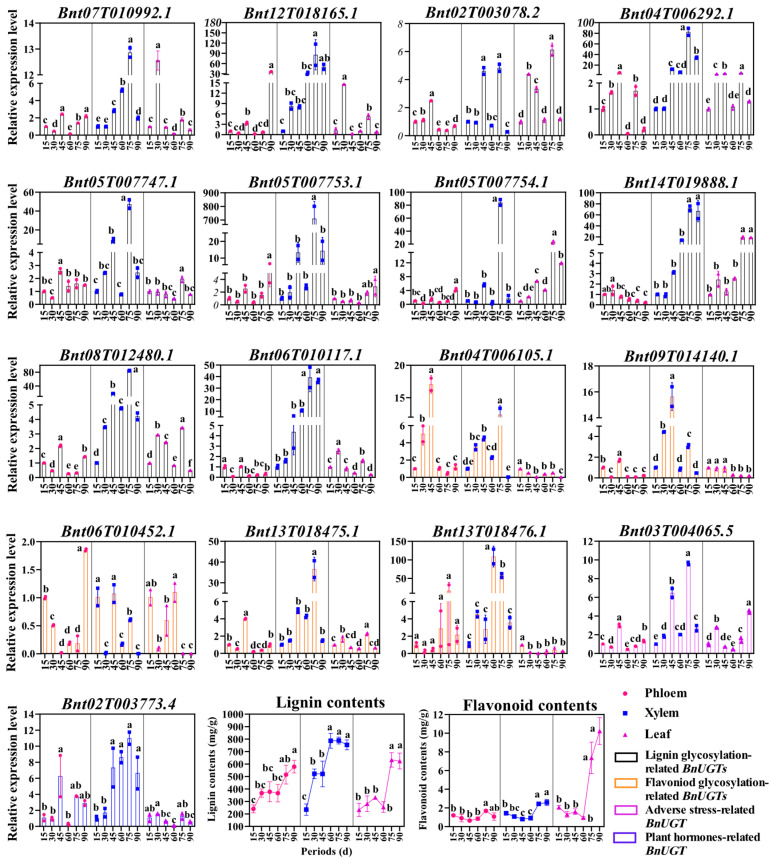
Expression profile of seventeen putative *BnUGTs*, lignin content, and flavonoid content in ramie during development. The column graph and line chart were made using the GraphPad Prism 8.0.2 software, which were edited using Adobe Illustrator 2020. The analysis was conducted via one-way analysis of variance (ANOVA) and Duncan’s multiple range test using the SPSS 26.0 software. Lowercase letters indicate significant differences.

The lignin content was upregulated in the phloem, xylem, and leaf during the six developmental periods (Figure 5). In the phloem, the lignin content was not significantly different between 15 and 60 days after emergence; after 75 days of emergence, the lignin accumulation was not significant, and the lignin content reached the maximum value, about 547 mg/g. However, the lignin content of the phloem was not significantly different between 30 and 75 days after emergence; the lignin content 90 days after emergence was significantly higher than that of other periods (except 75 days after emergence). In the leaf, the lignin content was not significantly different between 15 and 60 days after emergence. However, after 60 days of emergence, the lignin accumulation was rapid, and the lignin content reached the maximum value after 75 days of emergence, about 629 mg/g. In the xylem, the lignin content 15 days after emergence was significantly lower than that of other periods. The lignin content was not significantly different between 30 and 45 days after emergence. After 60 days of emergence, the lignin accumulation was significant, reaching a maximum value of about 776 mg/g, which was significantly higher than that of the phloem and leaf.

The flavonoid content showed varying trends across different tissues throughout the six developmental periods (Figure 5). In the phloem and leaf, the flavonoid content was not significantly different between 15 and 60 days after emergence, flavonoid accumulation was rapid after 60 days of emergence, and the flavonoid content reached the maximum value after 75 days of emergence, about 1.4 mg/g and 2.5 mg/g, respectively. Specifically, the flavonoid content was first downregulated and then upregulated in the xylem, and the flavonoid content reached the maximum value of about 8.8 mg/g after 75 days of germination. The content of flavonoids in the leaf was 6.3 times and 3.5 times that of the phloem and xylem after 75 days of germination, respectively.

The expression levels of seventeen putative *BnUGTs* were analyzed during the six developmental periods (Figure 5). In the phloem, eleven *BnUGT* expression patterns were observed; the most common expression pattern involved genes being downregulated and then upregulated, followed by further downregulation and then upregulation, including *Bnt07T010992.1*, *Bnt05T007754.1*, *Bnt08T012480.1*, *Bnt13T018475.1*, and *Bnt03T004065.5*. The expression level of *Bnt05T007753.1* was upregulated, the expression level of *Bnt14T019888.1* was downregulated, and the expression level of Bnt13T018476.1 was not significantly different between 15 and 90 days after emergence. In the leaf, seven BnUGT expression patterns were observed; the most common expression pattern involved genes being upregulated and then downregulated, followed by further upregulation and then downregulation, including *Bnt07T010992.1*, *Bnt12T018165.1*, *Bnt02T003078.2*, *Bnt04T006292.1*, *Bnt05T007754.1*, *Bnt08T012480.1*, *Bnt06T010117.1*, and *Bnt13T018475.1*. The expression levels of *Bnt05T007753.1* and *Bnt14T019888.1* were upregulated, and the expression levels of *Bnt04T006105.1*, *Bnt09T014140.1*, and *Bnt13T018476.1* were downregulated. In the xylem, four *BnUGT* expression patterns were observed; the most common expression pattern involved genes being upregulated and then downregulated, followed by further upregulation and then downregulation. This pattern has the same number of genes as the expression pattern, showing initial upregulation and then downregulation, with both containing six genes. The expression levels of *Bnt12T018165.1*, *Bnt14T019888.1*, *Bnt06T010117.1*, and *Bnt02T003773.4* were upregulated. The expression of monolignol-glycosylation-related candidate *BnUGTs* (except *Bnt07T010992*.1 and *Bnt02T003078*.2) in the xylem 75 days after germination was much higher than that during the other five periods. The relative expression ranged from 40 to 90, and the relative expression of *Bnt05T007753*.1 reached up to 710. The expression of *Bnt13T018475*.1 and *Bnt13T018476*.1 (flavonoid-glycosylation-related candidate *BnUGTs*) in the xylem 75 days after germination was much higher than that during the other five periods, and the relative expression of *Bnt13T018476.1* reached up to 109 at 60 days after germination.

### 2.7. Correlation Between Lignin Content and Putative BnUGT Expression

**Figure 6:**
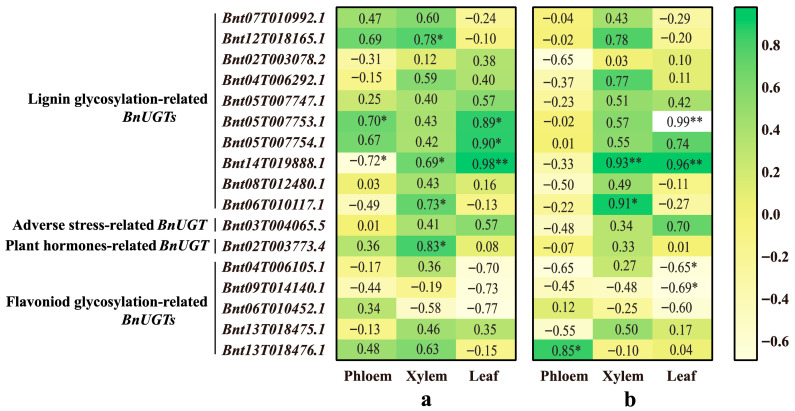
The heat map of the correlation between putative *BnUGT* expression, lignin content, and flavonoid content. (**a**) Correlation of lignin content with *BnUGT* expression. (**b**) Correlation of flavonoid content with *BnUGT* expression. The heat map was made using the GraphPad Prism 8.0.2 software and edited using Adobe Illustrator. The correlation analysis was conducted using the SPSS 26.0 software. The color scale is shown on the right. Green represents significant positive correlation, and faint yellow represents significant negative correlation. The data in the heat map represents the correlation coefficient. “**” Represents extreme significance; “*” represents significance.

The correlations between the expression levels of seventeen putative *BnUGT*s and lignin content in the phloem, xylem, and leaf during the six developmental stages were analyzed (Figure 6a). The lignin content was upregulated in the phloem, xylem, and leaf as the plant grew and developed; however, the expression levels of *BnUGT*s were different (Figure 5). In the phloem, only *Bnt05T007753.1* expression showed a significant positive correlation with lignin content, whereas *Bnt14T019888.1* expression showed a significant negative correlation with lignin content. In the xylem, the expression of *Bnt12T018165.1*, *Bnt14T019888.1*, *Bnt06T010117.1*, and *Bnt02T003773.4* showed a significant positive correlation with lignin content. In the leaf, the expression of *Bnt05T007753.1* and *Bnt05T007754.1* showed a significant positive correlation with lignin content, and *Bnt14T019888.1* expression showed an extremely significant positive correlation with lignin content.

### 2.8. Correlation Between Flavonoid Content and Putative BnUGT Gene Expression

The correlations between the expression levels of seventeen putative *BnUGT*s and flavonoid content in the phloem, xylem, and leaf during the six developmental stages were analyzed (Figure 6b). In the phloem, only *Bnt13T018476.1* expression showed a significant positive correlation with flavonoid content. In the xylem, *Bnt06T010117.1* expression showed a significant positive correlation with flavonoid content, and *Bnt14T019888.1* expression showed an extremely significant positive correlation with flavonoid content. In the leaf, the expression of *Bnt04T006105.1* and *Bnt09T014140.1* showed a significant negative correlation with flavonoid content, whereas the expression of *Bnt05T007753.1* and *Bnt14T019888.1* showed an extremely significant positive correlation with flavonoid content. It is worth noting that the expression of *Bnt06T010117.1* showed a strong positive correlation with lignin and flavonoid content in the xylem (Figure 6).

### 2.9. The Expression Patterns of Putative BnUGTs Under Cd Stresses

**Figure 7:**
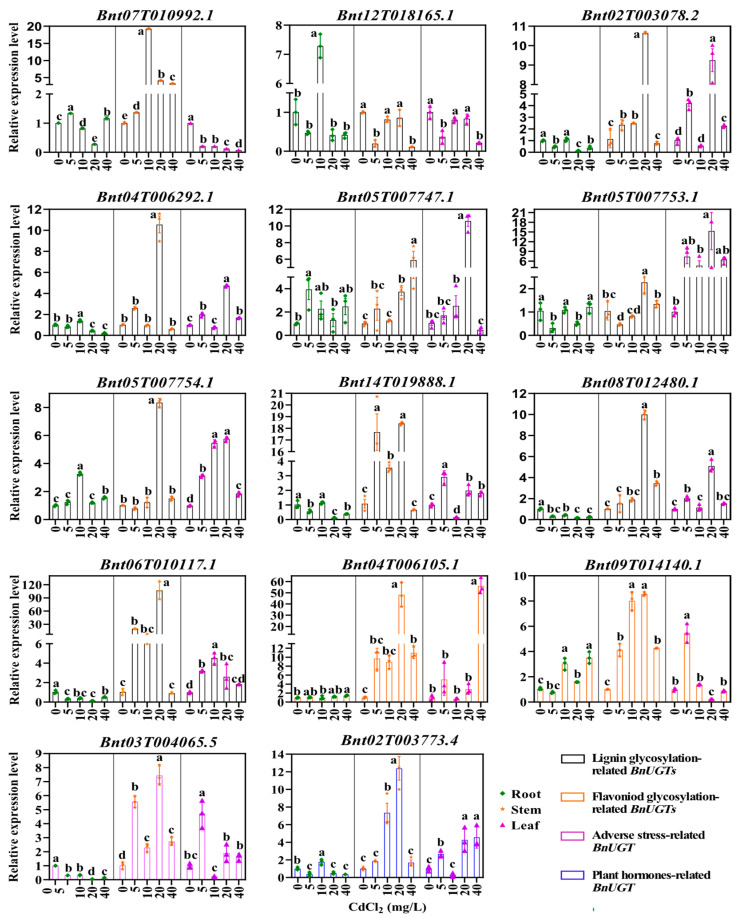
The expression profile of *BnUGT* genes in the root, stem, and leaf of ramie under Cd treatment. The column graph was made using the GraphPad Prism 8.0.2 software. The analysis was conducted via one-way analysis of variance (ANOVA) and Duncan’s multiple range test using the SPSS 26.0 software. Lowercase letters indicate significant differences.

To determine whether putative *BnUGT* genes were involved in abiotic stress responses, the expression of ten monolignol-glycosylation-related genes, two flavonoid-glycosylation-related *BnUGT* genes (*Bnt04T006105.1* and *Bnt09T014140.1*), and one adverse-stress-related *BnUGT* (*Bnt03T004065.5*) was analyzed using qRT-PCR (Figure 7). Eight *BnUGT* expression patterns were identified in ramie roots in response to increasing Cd stress. The expression of *Bnt04T006105.1* was upregulated, and that of *Bnt08T012480.1* and *Bnt03T004065.5* was downregulated. Four *BnUGT* expression patterns were observed in ramie stems in response to increasing Cd stress. Specifically, the expression of nine *BnUGT* genes was first upregulated and then downregulated; only the expression of *Bnt05T007747.1* was upregulated. The expression of *Bnt02T003078.2*, *Bnt04T006292.1*, *Bnt06T010117.1*, *Bnt04T006105.1*, and *Bnt02T003773.4* under 20 mg/L Cd treatment was much higher than at other Cd concentrations. In particular, the expression of *Bnt06T010117.1* increased 106-fold. Among the six *BnUGT* expression patterns in ramie leaves under increasing Cd stress, only the expression of *Bnt04T006105.1* and *Bnt07T010992.1* was upregulated and downregulated, respectively. The expression of *Bnt04T006105.1* under 40 mg/L Cd treatment was 10–50 times higher than at other Cd concentrations. Moreover, the expression of *Bnt02T003078.2*, *Bnt04T006292.1*, *Bnt05T007747.1*, *Bnt05T007753.1*, and *Bnt08T012480.1* under 20 mg/L Cd treatment was much higher than at other Cd concentrations. In addition, all genes had the lowest expression in the roots compared to stems and leaves (except *Bnt07T010992.1*), and the expression of *Bnt05T007753.1* in leaves was much higher compared to roots and stems.

## 3. Discussion

The *UGT* multigene family has been identified in several plant species, including eudicots and monocots. In total, 84 *BnUGT* family members were first identified in the *B. nivea* genome, and the number of *BnUGTs* in ramie is smaller than that of other plants, such as *A. thaliana* (107) [25], *P. trichocarpa* (192) [3], and *Camellia sinensis* (132) [37]. However, there was little difference in the number of UGTs in *Citrus sinensis* genes (Supplementary Table S4) [18]. The *UGTs* responsible for the glycosylation modification of monolignol are mainly distributed in Groups D, E, and L [35], in which 33 *BnUGT*s were identified in ramie. The *UGTs* responsible for the glycosylation modification of flavonoids are mainly distributed in Groups A, B, and F, in which 18 *BnUGT*s were identified in this study (Figure 2). Groups D and E are relatively large phylogenetic groups of plants whose members can recognize a range of substrates, such as terpenoids, flavonoids, benzoates, and lignin metabolism intermediates [25,32]. The number of *BnUGTs* in Groups D, E, and L (Supplementary Table S4) was higher compared to other groups, similar to other plants. Notably, Group O and Group P are newly discovered taxa in higher plants. Unlike *Arabidopsis* [25], these two groups also exist in ramie (Supplementary Table S4), which is consistent with poplar [3]. Groups Q and R were absent in *A. thaliana* [25], *P. trichocarpa* [3], and *Linum usitatissimum* L. [38]. Ramie and *C. sinensis* [37] were similar in the absence of Group Q. However, ramie contained one Group R member, which may be due to the distinct S/G/H lignin ratio in ramie (6:3:1) compared to that of model plants. As a fiber crop, members of Groups J and K were present in *L. usitatissimum* but not in ramie (Supplementary Table S4). This highlights lineage-specific gene loss or divergence and further underscores species-specific adaptations in UGT function [15].

Motifs are the locally conserved regions in the gene sequence and may play an essential role in specific biological functions. Conserved motifs are commonly regarded as key indicators for analyzing the expansion of gene families. Despite significant variation among these groups, motif 1, motif 2, and motif 4 exhibit high conservation in ramie *UGTs*, suggesting their potential importance in the glycosylation function of UGT enzymes, which is consistent with their role in substrate recognition [23]. This finding diverges from a previous report on *Ci. sinensis* (motif 1 and motif 3) [18]. The expansion of *UGT* families in ramie, particularly on chromosomes 4 and 6 (Figure 4), likely reflects gene duplication events that drive functional diversification. Moreover, we found three collinear gene pairs in this study, indicating that the arrangement order of *BnUGT*s on chromosomes was maintained. The number of *BnUGT* gene pairs in ramie is smaller than that of other plants, such as *Ci. sinensis* (27) [18], and all 191 *UGTs* in *Populus trichocarpa* were duplicated from each other [3]. This may indicate that *BnUGTs* in ramie have different biological functions than in other plants. These genomic features provide a foundation for further comparative studies aimed at elucidating the evolutionary dynamics of *UGT* genes in plants.

Monolignol glycosylation is essential for lignin biosynthesis and influences fiber quality and plant cell wall integrity. The lignin content was upregulated in the phloem, xylem, and leaf during the six developmental periods. Lignin accumulation was rapid after 60 days of emergence, and the lignin content reached the maximum value after 75 days of emergence in the phloem and leaf. This may be related to the functional requirements and physiological states of the phloem and leaf at different stages [39]. The phloem and leaf may focus more on nutrient transport between 15 and 60 days after emergence. The lignin content gradually increases after 60 days of emergence due to the demand for mechanical protection; thus, the best time for fiber harvest is before 75 days after emergence. Lignin content reaches its peak at 60 days after emergence, indicating rapid lignin accumulation in the xylem to establish a robust structure, facilitating mechanical support and water transport, which are necessary for rapid plant development [1]. Lignin accumulation gradually slows down after 60 days of emergence. The expression patterns of 17 putative *BnUGTs* were characterized in three tissues during six developmental stages, and the results indicated that *BnUGTs* were grouped into 11, 7, and 4 classes in the phloem, leaf, and xylem based on their expression profiles, respectively. *Bnt05T007753.1* expression showed a significant positive correlation with lignin content in the phloem and leaf. In addition, the expression level of *Bnt05T007753.1* in the xylem reached up to 710 after 75 days of germination. *Bnt14T019888.1* expression showed a significant negative correlation with lignin content in the phloem but a significant positive correlation with lignin content in the leaf and xylem. These results align with those of previous studies on *Arabidopsis* and poplar, where *UGT72* family members glycosylated monolignols to modulate lignin composition [7,10,11]. In addition, the gene module PdeWRKY65-UGT75L28 negatively regulates petiole lignification by modulating the glycosylation of coniferyl aldehydes in poplars [40]. We inferred that *Bnt05T007753.1 may* catalyze the glycosylation of caffeic acid [31] and subsequently participate in lignification, and *Bnt14T019888.1* simultaneously catalyzes the glycosylation of lignin monomers [32] and precursors (coniferyl aldehyde, sinapyl aldehyde, and sinapic acid) [10,33], thus reducing the amount of precursor material needed for lignin synthesis and consequently hampering lignin synthesis. Therefore, based on the phylogenetic tree, we further confirmed that *Bnt05T007753.1* and *Bnt14T019888.1* may play key roles in regulating lignin glycosylation during ramie growth.

Flavonoid glycosylation enhances solubility and stability and contributes to the medicinal properties of ramie [41]. The flavonoid content in the phloem and leaf during the six developmental periods was upregulated but showed no significant change from 15 to 60 days after germination. The flavonoid content significantly increased after 60 days of germination, which may be due to the rapid growth of seedlings as a result of consuming precursor substances, leading to flavonoid accumulation. However, the flavonoid content in the xylem was first downregulated and then upregulated during the six developmental periods. It is possible that the flavonoid was consumed during the initial growth [42]. The negative correlation between *Bnt04T006105.1*/*Bnt09T014140.1* expression and flavonoid content in leaves suggests complex regulatory mechanisms, possibly involving substrate competition or feedback inhibition, as observed in citrus flavonoid glycosyltransferases [16,18]. In addition, the evolutionary relationship suggests that *Bnt14T019888.1* and *Bnt06T010117.1* may participate in flavonoid and monolignol metabolism. We found that the expression of *Bnt06T010117.1* showed a significant positive correlation with both lignin and flavonoid content in the xylem. In the leaf and xylem, *Bnt14T019888.1* expression showed a significant positive correlation with both lignin and flavonoid content, respectively. Therefore, *Bnt14T019888.1* and *Bnt06T010117.1* may have the function of both *UGT75* and *UGT84* in *A. thaliana*. Specifically, the UGT glycosylates anthocyanin [34] and catalyzes the formation of cinnamic acid and hydroxycinnamic acids (*p*-coumaric acid, caffeic acid, ferulic acid, and sinapic acid) of lignin precursor glucose esters [35,36].

Glycosylation is a common modification in the synthesis of plant secondary metabolites and plays a critical role in normal plant growth and stress responses as an evolutionary mechanism [43,44]. This study revealed that *BnUGT* genes respond dynamically to Cd stress. *Bnt04T006105.1* (Group P) was upregulated in the stems and leaves under Cd treatment, thus suggesting its role in detoxification or stress signaling, similar to UGTs in *P. trichocarpa* that mitigate abiotic stress [3]. Citrus flavonoids are secondary metabolites that play crucial roles in the response to biotic and abiotic stresses, such as pathogen defense [38,45] and stress tolerance, and they have medicinal properties [18]. In the stem, the expression of *Bnt06T010117.1* increased 106-fold under 20 mg/L Cd treatment, and the expression of *Bnt06T010117.1* showed a significant positive correlation with both lignin and flavonoid content. Therefore, we inferred that *Bnt06T010117.1* may play a role in the response to stress by regulating lignin and flavonoid glycosylation in ramie. The presence of stress-responsive *cis*-elements, such as ABRE and TC-rich repeats, in *BnUGT* promoters further supported their involvement in stress adaptation. Additionally, *BnUGT*s in Group M (*Bnt02T003773.4*) and P were linked to hormone glycosylation (e.g., cytokinins) [24], which may influence growth and stress responses. For example, *Bnt02T003773.4* expression in the stem under 20 mg/L Cd treatment was much higher than that under other Cd concentrations.

Four candidate *BnUGT*s (*Bnt05T007753.1*, *Bnt14T019888.1*, *Bnt06T010117.1*, and *Bnt04T006105.1*) were screened from the genome of ramie in this study. However, further enzymatic assays and functional validation of these four candidate *BnUGT*s are needed to further confirm their roles in lignin/flavonoid glycosylation and stress responses.

## 4. Materials and Methods

### 4.1. Plant Materials, Sample Preparation, and Abiotic Stress Treatment

Wild ramies were planted at the Hunan University of Arts and Science (29° N and 111° E). The ramies were planted in the field for stump propagation and routine management. The stem segment of the middle part and 4–6 leaves below the top bud of the wild ramies were harvested at 15, 30, 45, 60, 75, and 90 d after germination and were used as the study materials (Supplementary Figure S2). Three randomly selected plants with the same growth tendencies were cut into small 1 cm pieces. The bark (phloem) and stalk (xylem) of the stems were separated using the method described by Tang et al. [46] (Supplementary Figure S2). After mixing the three samples, part of the samples were quickly frozen in liquid nitrogen and stored at −80 °C for total RNA extraction. The remaining samples were dried in an oven at 60 °C and ground to a powder in a tissue grinding mill MM 400 (RETSCH, Haan, Germany). The powder was sieved through a 30-mesh screen to determine the lignin and flavonoid contents according to the method described by Tang et al. [47]. Three biological replicates were used for each experiment, and two technical replicates were used for each biological replicate.

Ramie seedlings were treated with different CdCl_2_ concentrations according to the method described by Tang et al. [47]; the Cd^2+^ concentrations were 0, 5, 10, 20, and 40 mg/L. The roots, stems, and leaves of three ramie seedlings were randomly selected after treatment for 48 h with CdCl_2_, mixed, flash-frozen in liquid nitrogen, and stored at −80 °C until total RNA extraction. Three replicates were analyzed for each treatment group.

### 4.2. Identification and Characterization of UGT Family Members in B. Nivea

*Boehmeria nivea* reference genome data (GCA_0181312145.1) [48] were obtained from the National Center for Biotechnology Information (NCBI). To identify putative *UGT* genes in ramie, the hidden Markov model file corresponding to the UGT domain (PF00201) was retrieved from the Pfam database and used as a query to perform an hmmsearch against ramie protein sequences using the online HMMER tool. Simultaneously, a local Basic Local Alignment Search Tool search between ramie and *A. thaliana* UGT protein sequences was conducted. The presence of the conserved PGPS domain within the obtained protein sequences was confirmed using the NCBI Batch CD-Search Tool (*E*-value < 0.01). Finally, 84 genes were characterized as *UGT* genes in *B. nivea* (*BnUGT*) based on the above analyses. The coding and protein sequences of *BnUGT* genes were obtained from a genomic annotation file. Physicochemical properties, such as the theoretical isoelectric point (pI), grand average of hydropathicity (GRAVY), index values, and molecular weights (MW) of *BnUGT* genes, were analyzed using the ProtParam tool (https://web.expasy.org/protparam/, accessed on 10 April 2025). The subcellular localization of *BnUGT* genes was predicted using the Plant-mPLoc tool.

### 4.3. Multiple Sequence Alignment, Phylogenetic Analysis, and Classification of BnUGT Genes

In total, 90 *UGT* protein sequences from *A. thaliana* (Groups A–N) [23], 138 from *Populus trichocarpa* Torr. & A.Gray ex Hook. (Groups A–P) [3], 3 from *Populus tomentosa* Carrière (Group E), 17 from *P. bretschneideri* (Groups D–L, O–P) [12], 11 from *Zea mays* L. (Groups A, M, O–Q) [24], and 2 from *C. sinensis* (Groups D and R) [43] were used to establish the neighbor-joining phylogenetic tree with ramie *UGT* proteins using the MEGA11 software (1000 bootstrap). The *UGT* gene sequences of these plants are presented in Supplementary Tables S2 and S3. Multiple protein alignments between *BnUGT* genes and other plant *UGT* genes were performed using MUSCLE with default parameters. A phylogenetic tree was generated using TBtools-II v2.154.

### 4.4. BnUGT Gene Structure and Conserved Motif Analysis

The gene structures of *BnUGT* genes were characterized based on the *B. nivea* genome annotation file. Conserved motifs of *BnUGT* genes were identified using the MEME online tool. The parameters were as follows: maximum number of motifs = 12, maximum motif width = 100, and minimum motif width = 50. The final gene structures and compositions of the conserved motifs were visualized using TBtools.

### 4.5. Chromosomal Distribution, Gene Collinearity, and Cis-Element Analysis of BnUGT Genes

The chromosome location of the *BnUGT* genes was visualized with the *B. nivea* genome annotation file using the advanced Circos of TBtools. The MCscanX program was used to analyze tandem and segmental duplication and collinearity within species. These results were visualized using TBtools. Cis-element analysis of ramie *UGT* genes located 2.0 kb upstream of ATG was performed. The cis-element composition analysis of *BnUGT* genes was conducted in PlantCARE, including six types, their numbers, and positions.

### 4.6. Expression Profile Analysis of BnUGT Genes

Primer 5.0 was used to design primers (Supplementary Table S7) within the conserved domain database sequence regions based on the nucleotide sequences of the *BnUGT* genes. Total RNA extraction and cDNA synthesis were performed as described by Tang et al. [47]. The quantitative real-time PCR (qRT-PCR) reaction system contained 10 μL of 2 × ChamQ Universal SYBR qPCR Master Mix (Vazyme, Nanjing, China), 1.0 μL of cDNA, 0.4 μL of Primer-P1, and 0.4 μL of Primer-P2. Sterile water was added to ensure a total volume of 20 μL, and the cDNA concentration was 750 ng/µL. The amplification reaction program was as follows: 95 °C for 30 s, followed by 40 cycles of 95 °C for 10 s and 60 °C for 30 s. The melting curve program was as follows: 95 °C for 15 s, 60 °C for 60 s, and 95 °C for 15 s using the LightCycler 480 Software release 1.5.0 (Roche, Basel, Switzerland). Three biological replicates were used for each experiment, and two technical replicates were used for each biological replicate. Considering actin-1 as the reference gene [49], the expression level of each gene was determined by analyzing data using the 2^−ΔΔCt^ method.

### 4.7. Statistical Analysis

The pictures were edited using Adobe Illustrator (version 20; Adobe, Dublin, UK) 2020. The column graph and line chart were made using the GraphPad Prism 8.0.2 software, which were edited using Adobe Illustrator. Statistical significance analysis of all data was performed using the SPSS software (version 26.0, Chicago, IL, USA) via one-way analysis of variance (ANOVA) and Duncan’s multiple range test. Subsequently, gene expression levels, lignin content, and flavonoid content were analyzed via bivariate correlations using the SPSS software. The results of the one-way ANOVA are labeled using letters. Lowercase letters indicate significant differences.

## 5. Conclusions

We identified 84 *BnUGT* family members in the *B. nivea* genome. The phylogenetic relationships and gene structures of these *BnUGTs* were systematically characterized. They all contained the PSPG-box and could be divided into 15 groups. Ten putative monolignol-glycosylation-related *BnUGT*s, five putative flavonoid-glycosylation-related *BnUGT*s, one putative adverse-stress-related *BnUGT*, and one putative plant-hormone-related *BnUGT* were identified through phylogenetic tree cluster analysis. Through further analysis of the expression, correlation, and stress of responses of 17 putative *BnUGTs*, we identified 4 candidate *BnUGTs*, including *Bnt05T007753.1*, which may participate in monolignol glycosylation; *Bnt14T019888.1* and *Bnt06T010117.1*, which may simultaneously participate in flavonoid and monolignol glycosylation; *Bnt04T006105.1*, which may respond to abiotic stress in ramie; and *Bnt06T010117.1*, which may play a role in stress through regulating lignin and flavonoid glycosylation in ramie. These findings contribute to a comprehensive understanding of glycosylation mechanisms and stress responses in ramie.

## Data Availability

All data reported in this study can be found in the manuscript file. Publicly available genome data GCA_0181312145.1 can be found in the NCBI database.

## Supplementary Materials

The following supporting information can be downloaded at: https://www.mdpi.com/article/10.3390/plants14162517/s1, Figure S1: Discovered motifs and motif locations; Figure S2: Ramie at 0, 15, 30, 45, 60, 75, and 90 days after germination; Table S1: Characterization of *BnUGTs* in ramie; Table S2: Protein sequence of *UGTs* in *Boehmeria nivea*; Table S3: Protein sequence of *UGTs* in other plants; Table S4: Comparison of ramie *UGT* phylogenetic groups with those of other published plants; Table S5: The conserved domain analysis of BnUGT proteins; Table S6: Analysis of cis-acting elements of BnUGTs; Table S7: Primers used in the qRT-PCR of *BnUGT* genes.
