# Supplementary material for: Genome-Wide Analysis and Screening of Uridine Diphosphate-Glycosyltransferase Family Genes Involved in Lignin/Flavonoid Glycosylation and Stress Response in Boehmeria nivea (L.) Gaudich
Source: Plants (Basel). 2025 Aug 13;14(16):2517. doi: 10.3390/plants14162517 (PMC12389536; doi:10.3390/plants14162517)
Supplement: Supplementary file 1 [file plants-14-02517-s001.zip › plants-3768314-supplementary.pdf]

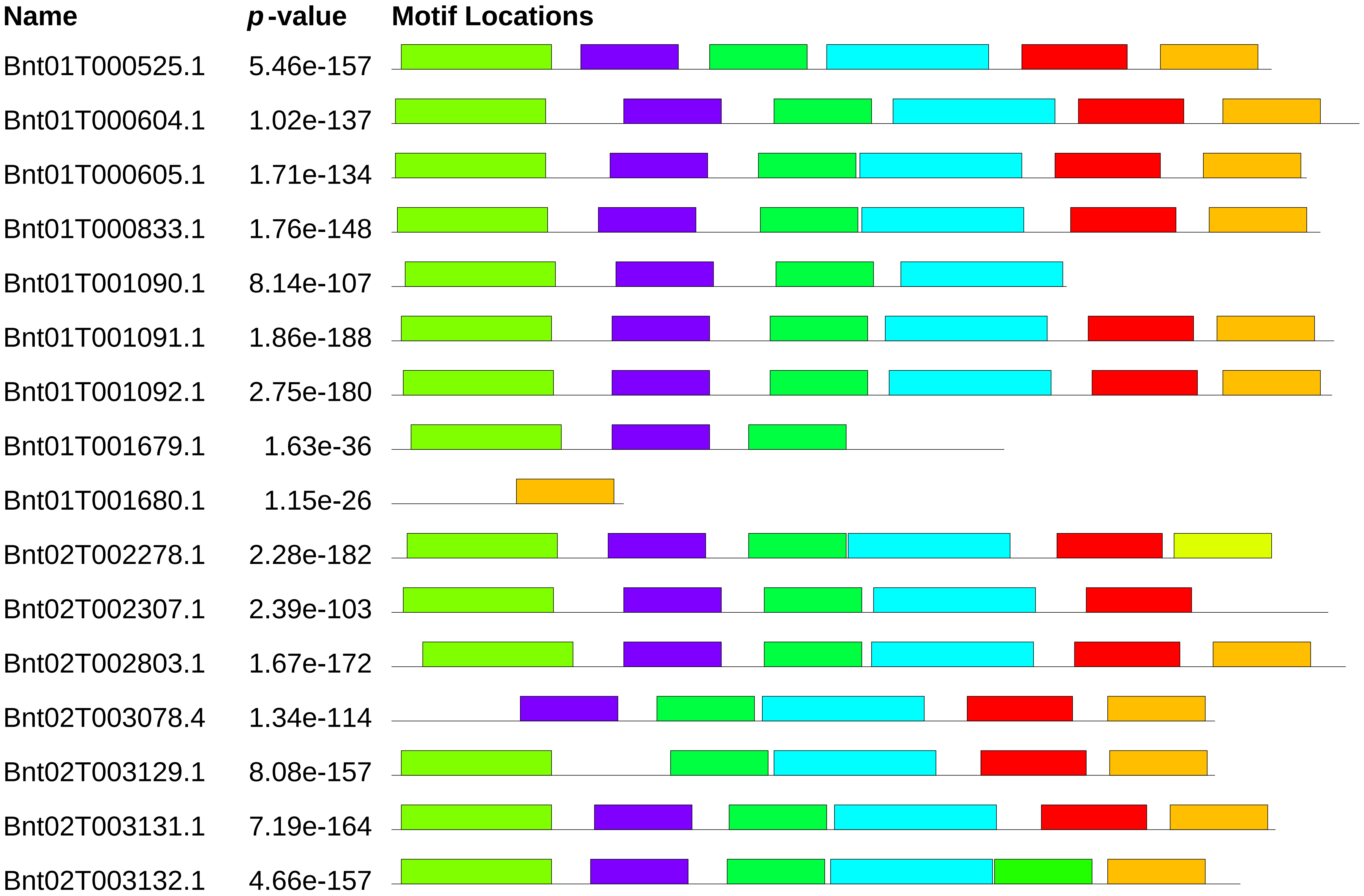
Name
p-value
Motif Locations
Bnt01T000525.1 5.46e-157
Bnt01T000604.1 1.02e-137
Bnt01T000605.1 1.71e-134
Bnt01T000833.1 1.76e-148
Bnt01T001090.1 8.14e-107
Bnt01T001091.1 1.86e-188
Bnt01T001092.1 2.75e-180
Bnt01T001679.1 1.63e-36
Bnt01T001680.1 1.15e-26
Bnt02T002278.1 2.28e-182
Bnt02T002307.1 2.39e-103
Bnt02T002803.1 1.67e-172
Bnt02T003078.4 1.34e-114
Bnt02T003129.1 8.08e-157
Bnt02T003131.1 7.19e-164
Bnt02T003132.1 4.66e-157

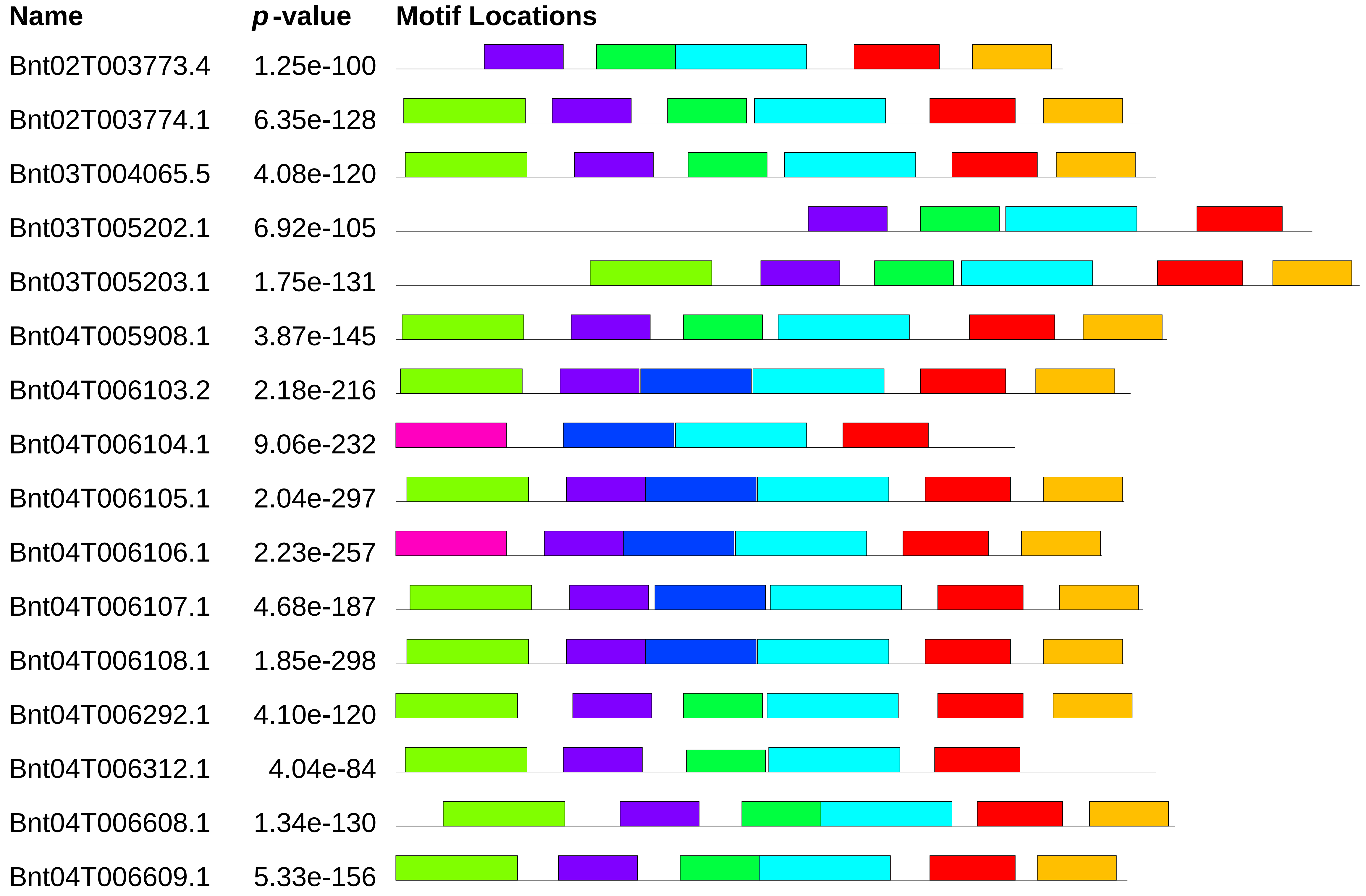
Name
p-value
Motif Locations
Bnt02T003773.4 1.25e-100
Bnt02T003774.1 6.35e-128
Bnt03T004065.5 4.08e-120
Bnt03T005202.1 6.92e-105
Bnt03T005203.1 1.75e-131
Bnt04T005908.1 3.87e-145
Bnt04T006103.2 2.18e-216
Bnt04T006104.1 9.06e-232
Bnt04T006105.1 2.04e-297
Bnt04T006106.1 2.23e-257
Bnt04T006107.1 4.68e-187
Bnt04T006108.1 1.85e-298
Bnt04T006292.1 4.10e-120
Bnt04T006312.1 4.04e-84
Bnt04T006608.1 1.34e-130
Bnt04T006609.1 5.33e-156

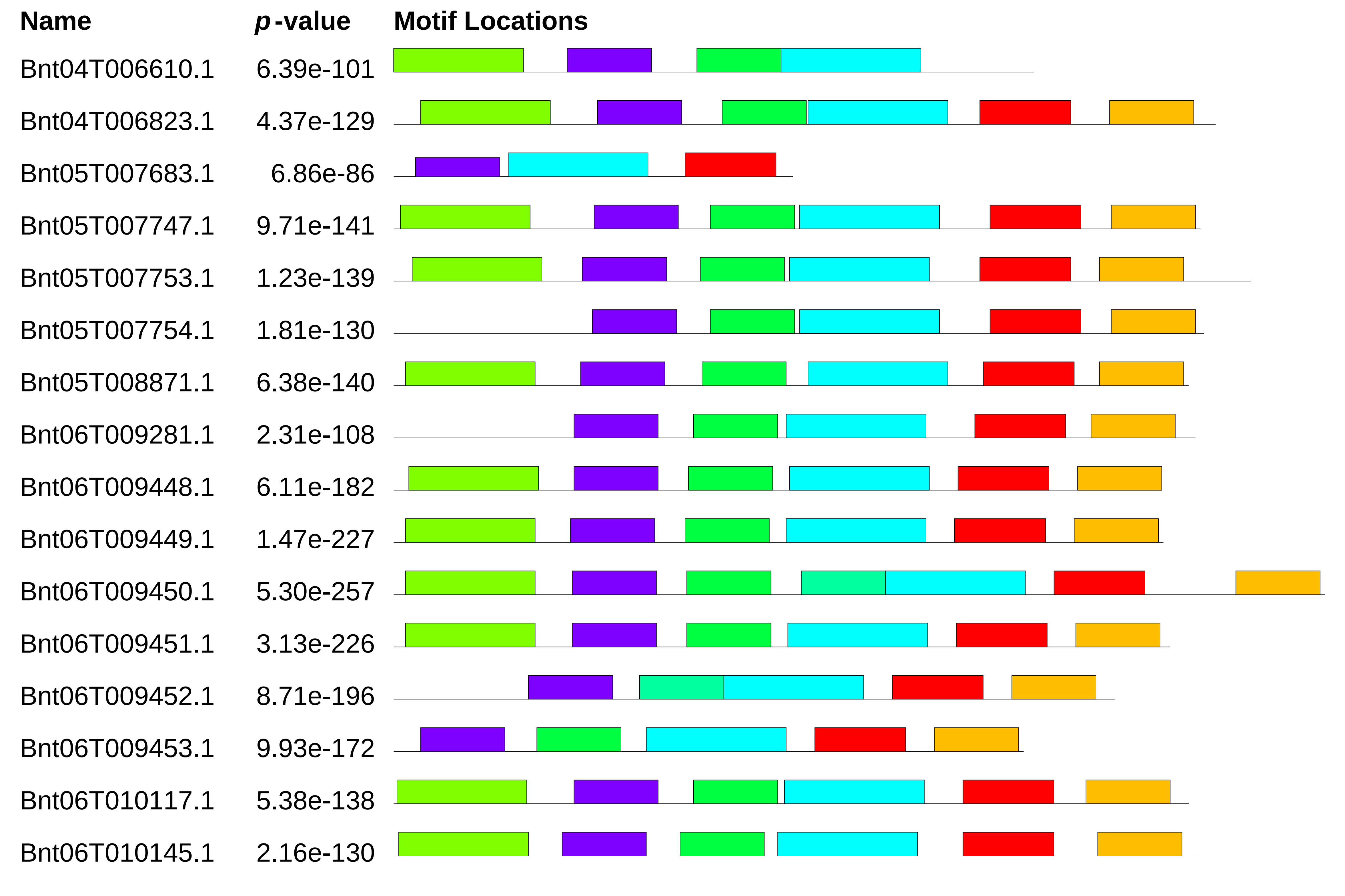

Name
p-value
Motif Locations
Bnt04T006610.1 6.39e-101
Bnt04T006823.1 4.37e-129
Bnt05T007683.1 6.86e-86
Bnt05T007747.1 9.71e-141
Bnt05T007753.1 1.23e-139
Bnt05T007754.1 1.81e-130
Bnt05T008871.1 6.38e-140
Bnt06T009281.1 2.31e-108
Bnt06T009448.1 6.11e-182
Bnt06T009449.1 1.47e-227
Bnt06T009450.1 5.30e-257
Bnt06T009451.1 3.13e-226
Bnt06T009452.1 8.71e-196
Bnt06T009453.1 9.93e-172
Bnt06T010117.1 5.38e-138
Bnt06T010145.1 2.16e-130

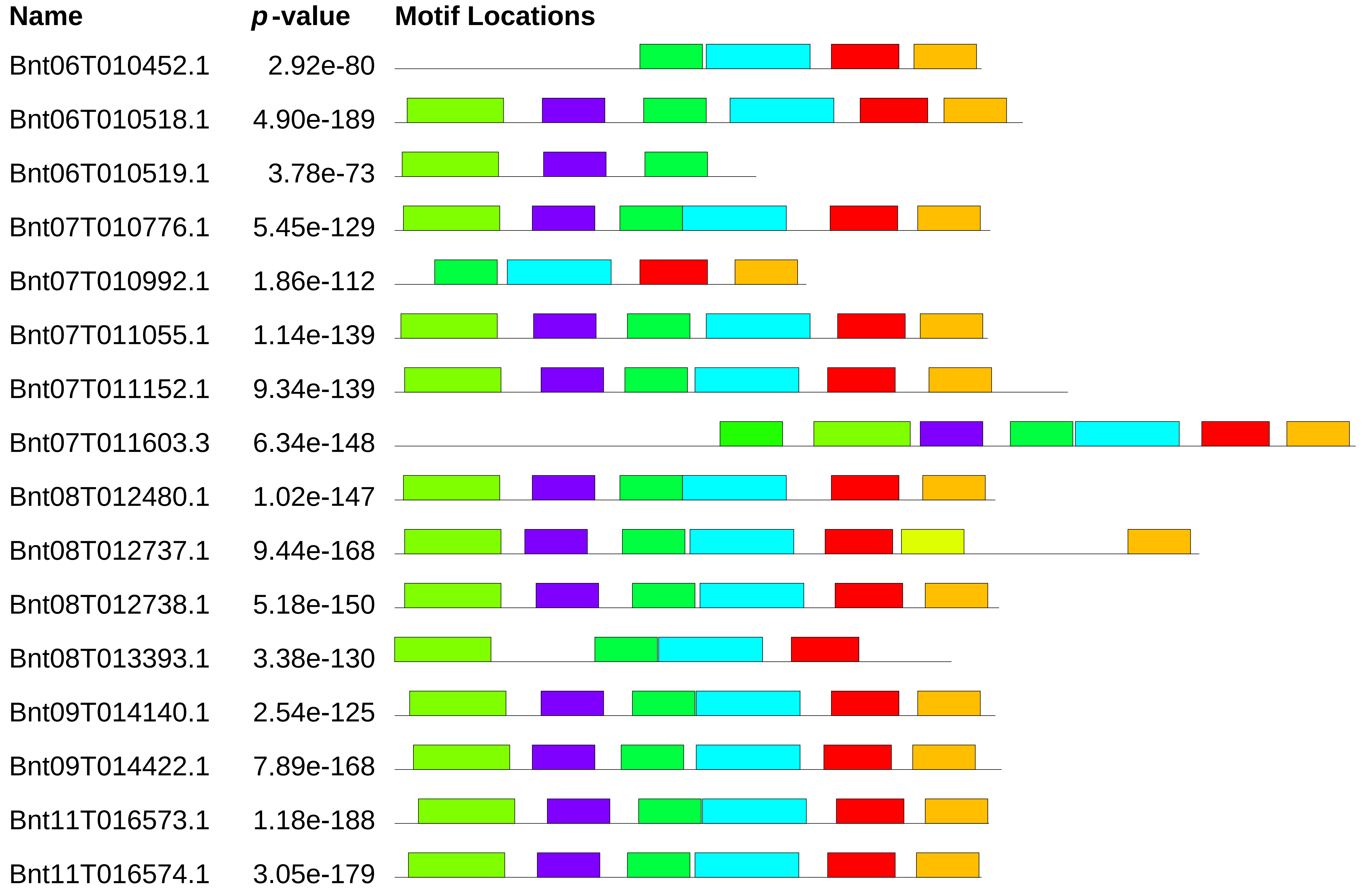
Name
p-value
Motif Locations
Bnt06T010452.1 2.92e-80
Bnt06T010518.1 4.90e-189
Bnt06T010519.1 3.78e-73
Bnt07T010776.1 5.45e-129
Bnt07T010992.1 1.86e-112
Bnt07T011055.1 1.14e-139
Bnt07T011152.1 9.34e-139
Bnt07T011603.3 6.34e-148
Bnt08T012480.1 1.02e-147
Bnt08T012737.1 9.44e-168
Bnt08T012738.1 5.18e-150
Bnt08T013393.1 3.38e-130
Bnt09T014140.1 2.54e-125
Bnt09T014422.1 7.89e-168
Bnt11T016573.1 1.18e-188
Bnt11T016574.1 3.05e-179

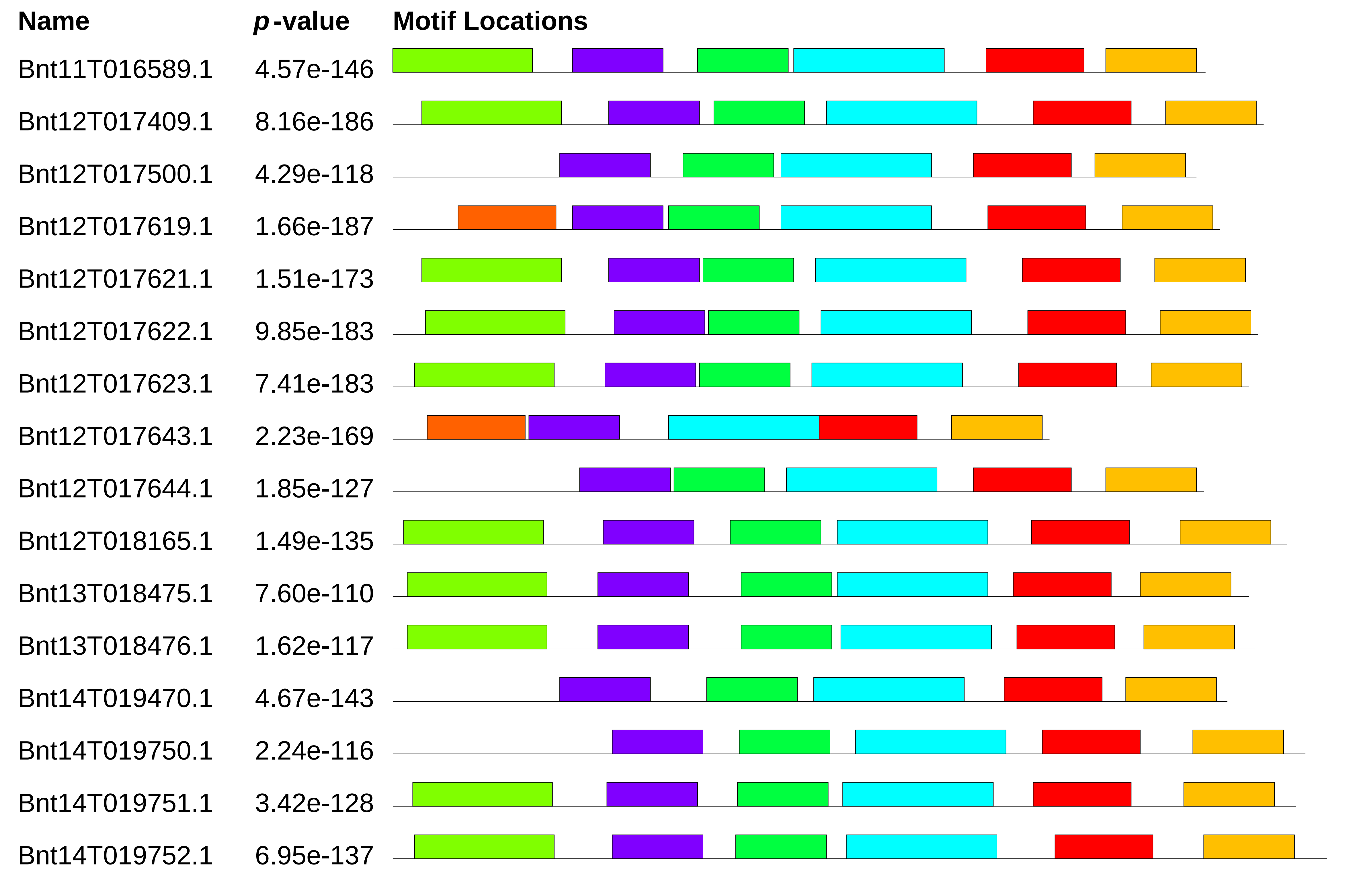
Name
p-value
Motif Locations
Bnt11T016589.1 4.57e-146
Bnt12T017409.1 8.16e-186
Bnt12T017500.1 4.29e-118
Bnt12T017619.1 1.66e-187
Bnt12T017621.1 1.51e-173
Bnt12T017622.1 9.85e-183
Bnt12T017623.1 7.41e-183
Bnt12T017643.1 2.23e-169
Bnt12T017644.1 1.85e-127
Bnt12T018165.1 1.49e-135
Bnt13T018475.1 7.60e-110
Bnt13T018476.1 1.62e-117
Bnt14T019470.1 4.67e-143
Bnt14T019750.1 2.24e-116
Bnt14T019751.1 3.42e-128
Bnt14T019752.1 6.95e-137

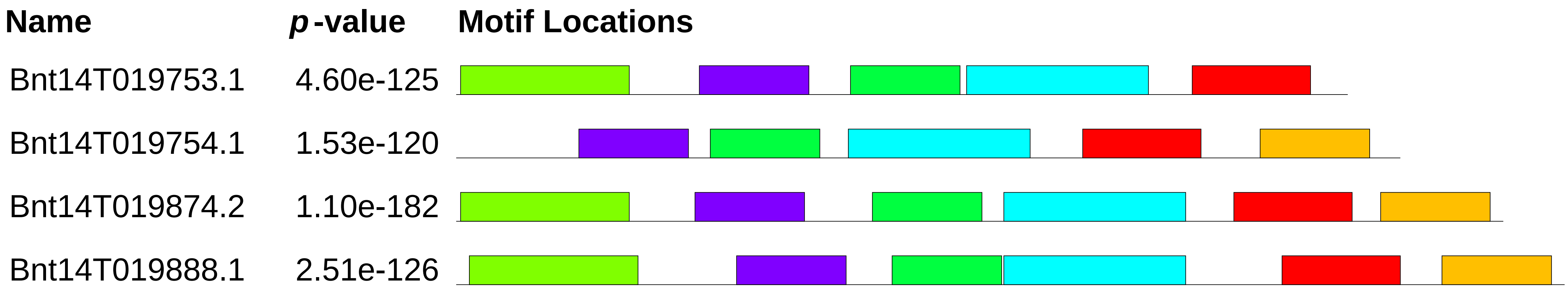

Motif	Symbol	Motif Consensus
1.		WAPQLEILAHPSVGGFVTHCGWNSTLESLSAGVPMVAWPLFADQFTNARLVVDV
2.		PTPPPPLPGLVLLSDGPSSSSSDSKDEEKCLEWLDSQEPKSVVYVSFGSLVTLSAEQLREJALGLERSGQPFLWVVRPPEGAE
3.		SSKPHIVMVPFPAQGHINPMLQLAKLLASKGFKVTFVVTPFNSPRLQKSASFPSPSGNPLVRVLLPPDGLPPAALAD
4.		RGARVDCVVSDAFMSWALDVARELGIPRAAFFTSSAAVLAAYLSLPKLLL
5.		EEVEEVVRELMEGEEGKEMRRRAKELKELARKAVEEGGSSDENLDELVAE
6.		PEDLPSLPSTLDSBDDDYSFLLRQASSLRKADGVLVNTFRELEPEYLDLJ
7.		GKLSEDDLMQPPPGFPDSSITLRRHEARASIGYRTTKFGGDVYFIDRIFTGLSECDAIGFKACREIDGPF
8.		MYPWFAIGHITPYLHFANKLAQKGHKISFLIPTKTQLKFHHMNLFPDLITFVPITVPHVDGLPPGAETTY
9.		SQRKSRAANRNPNPNIVFHALPAPEVAPSPSPDSNGFAAHFVPSFEASIGLRRP
10.		PRDEKDGSFTRDLVAESJKRVMEDEEGEIIREKAKEMREIFCDRELQDKM
11.		DWIEQLGEGFHNAVCEEGCLVKWEPGKDIGAEGREQYPNVRYASVEWRMG
12.		LKPDDLPLFIRDPGLCPSFLKMVNEQFDWLDGPDWEVDWIAKILRLRTIG

Motif	E-value	Sites	Width
Motif 1	5.6e-2477	78	54
Motif 2	2.5e-1855	81	83
Motif 3	1.2e-901	64	77
Motif 4	2.8e-788	78	50
Motif 5	3.4e-692	72	50
Motif 6	7.9e-445	74	50
Motif 7	6.3e-151	6	70
Motif 8	7.5e-035	2	70
Motif 9	3.2e-002	2	54
Motif 10	8.2e+001	2	50
Motif 11	5.3e+004	2	50
Motif 12	8.3e+004	2	50

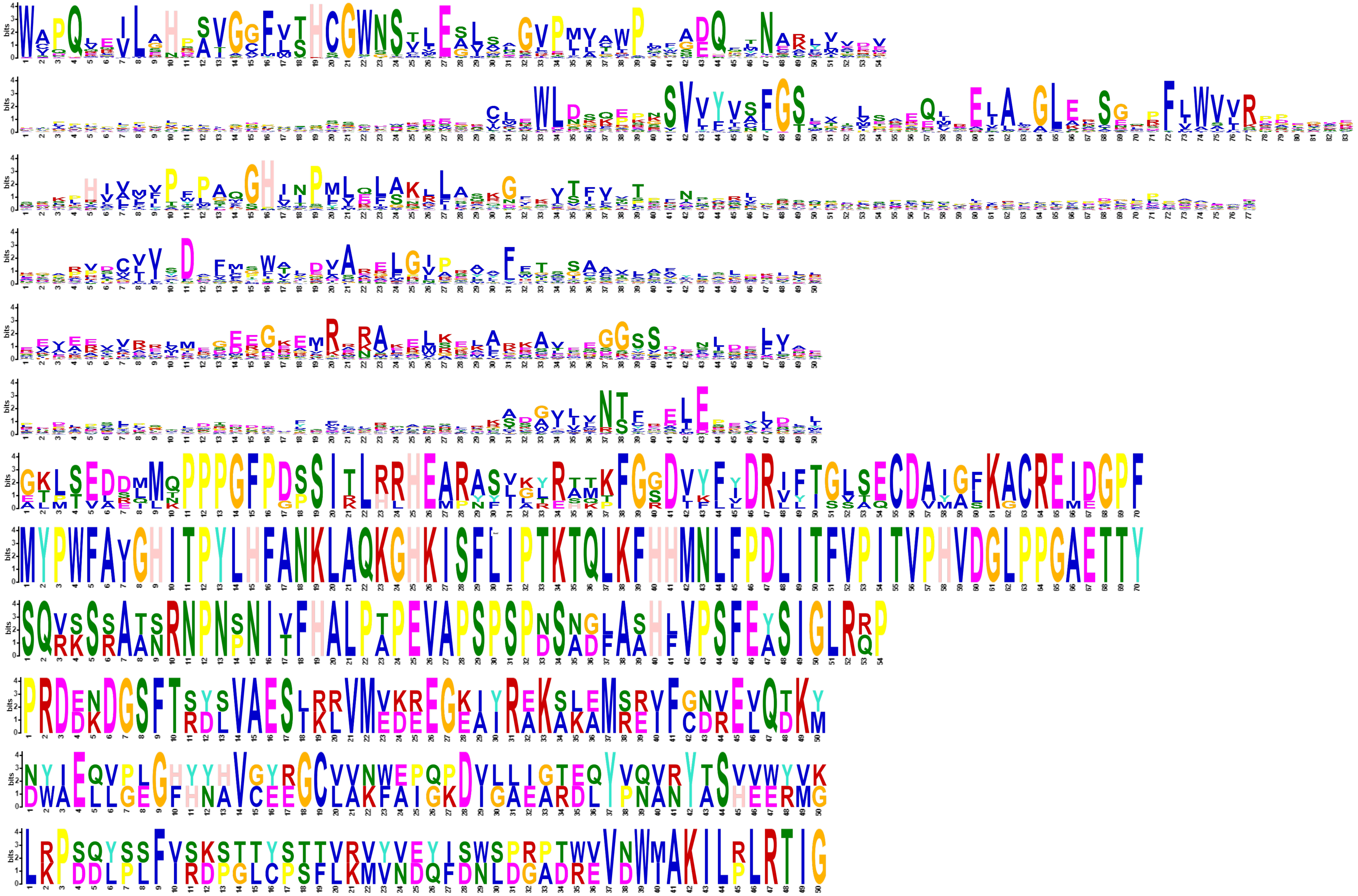

Figure S1. Discovered motifs and motif locations.

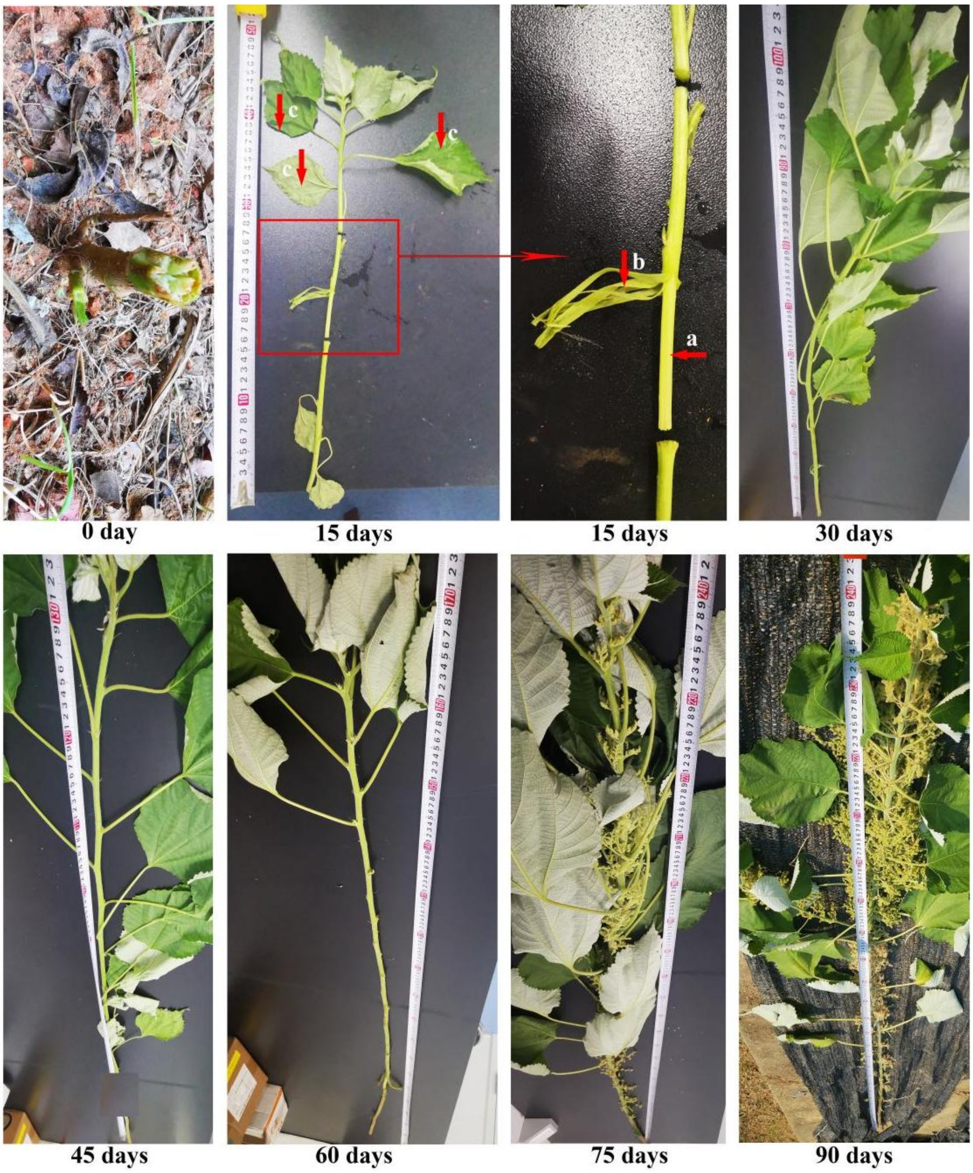

Figure S2. Ramie at 0, 15, 30, 45, 60, 75 and 90 days after germinate. Red boxes indicate the stem segment of the middle part; a indicate stem xylem; b indicate Stem phloem; c indicate the 4-6 leaves below the top bud.
